# Cold EMR vs. Hot EMR for the removal of sessile serrated polyps larger than 10 mm: a systematic review and meta-analysis

**DOI:** 10.1186/s12893-024-02325-2

**Published:** 2024-03-20

**Authors:** Cong Ding, Jian-feng Yang, Xia Wang, Yi-feng Zhou, Hayat Khizar, Zheng Jin, Xiao-feng Zhang

**Affiliations:** 1 Department of Gastroenterology, Affiliated Hangzhou First People’s Hospital, School of Medicine, Westlake University, Hangzhou, Zhejiang Province China; 2 Key Laboratory of Clinical Cancer Pharmacology and Toxicology Research of Zhejiang Province, Hangzhou, Zhejiang Province China

**Keywords:** Sessile serrated polyps, Endoscopic mucosal resection, Adverse event, Meta-analysis

## Abstract

**Background:**

Endoscopic mucosal resection (EMR) appears to be a promising technique for the removal of sessile serrated polyps (SSPs) ≥ 10 mm. To assess the effectiveness and safety of EMR for removing SSPs ≥ 10 mm, we conducted this systematic review and meta-analysis.

**Methods:**

We conducted a thorough search of Embase, PubMed, Cochrane, and Web of Science databases for relevant studies reporting on EMR of SSPs ≥ 10 mm, up until December 2023. Our primary endpoints of interest were rates of technical success, residual SSPs, and adverse events (AE).

**Results:**

Our search identified 426 articles, of which 14 studies with 2262 SSPs were included for analysis. The rates of technical success, AEs, and residual SSPs were 100%, 2.0%, and 3.1%, respectively. Subgroup analysis showed that the technical success rates were the same for polyps 10–19 and 20 mm, and en-bloc and piecemeal resection. Residual SSPs rates were similar in en-bloc and piecemeal resection, but much lower in cold EMR (1.0% vs. 4.2%, *P* = 0.034). AEs rates were reduced in cold EMR compared to hot EMR (0% vs. 2.9%, *P* = 0.168), in polyps 10–19 mm compared to 20 mm (0% vs. 4.1%, *P* = 0.255), and in piecemeal resection compared to en-bloc (0% vs. 0.7%, *P* = 0.169).

**Conclusions:**

EMR is an effective and safe technique for removing SSPs ≥ 10 mm. The therapeutic effect of cold EMR is superior to that of hot EMR, with a lower incidence of adverse effects.

**PROSPERO registration number:**

CRD42023388959.

**Supplementary Information:**

The online version contains supplementary material available at 10.1186/s12893-024-02325-2.

## Introduction

Colorectal cancer (CRC) is the third most common cancer in males and the second most prevalent cancer in females worldwide. It is also responsible for the third-largest number of cancer-related deaths worldwide [1]. The development of colorectal cancer is caused by mutations in genes that occur via one of three distinct pathways: the chromosomal instability pathway, the microsatellite instability pathway, or the serrated pathway. Each of these pathways leads to the development of preneoplastic lesions, which include tubular/villous adenomas and serrated lesions [2]. It is believed that serrated polyps, which derive their name from their distinctive “saw-toothed” histological appearance, progress to colorectal cancer via the serrated pathway [3]. According to the criteria established by the World Health Organization, serrated polyps can be pathologically categorized as hyperplastic polyps (HPs), sessile serrated adenoma/polyps (SSA/Ps) with or without cytological dysplasia, or classic serrated adenomas (TSAs). Recent studies suggest that sessile serrated adenomas/polyps (SSA/Ps) constitute the primary precursor lesion of serrated pathway cancers. Serrated pathway cancers account for 20–30% of all spontaneous cases of CRC and are a significant contributor to the incidence of CRC [2, 4–6].

Polyps that are 6 to 9 mm in size and micro polyps measuring 1 to 5 mm typically lack the features associated with advanced adenomas when compared to larger polyps (≥ 10 mm). Therefore, it is necessary to surgically remove large polyps (≥ 10 mm) [7]. European Society of Gastrointestinal Endoscopy Cascade Guideline recommends cold snare polypectomy (CSP) as the preferred technique for removal of diminutive polyps (size ≤ 5 mm) and suggests CSP for sessile polyps 6–9 mm in size because of its superior safety profile [8]. But polyps 10–19 mm in diameter are safer and less difficult to be removed by endoscopic mucosal resection (EMR). Polyps ≥ 20 mm are more difficult to remove with EMR and are treated with endoscopic submucosal dissection [9]. EMR is a minimally invasive endoscopic technique employed for the removal of colorectal tumors. This procedure entails the injection of submucosal fluid to create a wider separation between the mucosa and submucosa, followed by a one-time or piecemeal snared polypectomy. In cases where accurate identification of the intestinal wall is challenging and poses a risk of bleeding during the excision process, a block excision may be utilized [10]. However, it is currently unknown which method of excision of serrated polyps greater than 10 millimeters in size is the most effective. Recent studies have been conducted on the efficacy and safety of endoscopic resection for SSPs, including comparisons of CSP to hot snare polypectomy (HSP) and cold snare polypectomy to cold EMR (c-EMR) [11, 12]. The technique of conventional EMR is the application of heat transmission to aid in resection and ablation, known as hot EMR (h-EMR) [13]. However, electropermeability is the main reason for the increased risk of delayed hemorrhage and perforation associated with EMR. C-EMR is an alternative to h-EMR because of its advantage in reducing the incidence of adverse events [14]. But the polyp recurrence rate is higher with c-EMR [15] Therefore, there is a strong need for a comprehensive assessment of the safety of cold and hot EMR for removing SSPs ≥ 10 mm. However, these studies have limitations, such as a very small sample size and predominantly reporting experiences from a single center [16–21].

## Aim

To report on the efficacy and safety of EMR for SSPs measuring 10 millimeters in size, we conducted this systematic review and meta-analysis.

## Materials and methods

### Search strategy

This systematic review and meta-analysis adheres to the Preferred Reporting Items for Systematic Reviews and Meta-Analyses (PRISMA) Statement and was registered with PROSPERO International Prospective Register of Systematic Reviews (registration number: CRD42023388959). The literature search was conducted on December 8, 2023 using medical databases such as Embase, PubMed, Cochrane, and Web of Science. The full search strategy for PubMed is available in Table S1. In addition, we screened the reference lists of eligible studies and relevant reviews to identify any additional studies that met our inclusion criteria. The search results were managed using citation software, Endnote.

### Inclusion and exclusion criteria

We included the studies in this systematic review and meta-analysis that meet the following criteria:

1. Studies reporting efficacy or safety data on EMR for the treatment of SSPs;
2. Full-length articles published in English;
3. Studies involving human subjects.

We excluded the studies in this systematic review and meta-analysis that meet the following criteria:

1. Studies that do not report outcomes of interest;
2. Abstracts, case reports, or case series with less than 10 patients;
3. Studies reporting outcomes on SSPs smaller than 10 mm;
4. Studies in other languages or animal studies.

### Study selection

Two reviewers independently screened the titles and abstracts of all papers resulting from the pre-specified search. Studies meeting the inclusion criteria were retrieved for full-text review and any reasons for exclusion were documented. Discrepancies between the two reviewers were resolved through discussion and consensus, with the involvement of a third investigator if necessary.

### Data extraction and quality assessment

Two reviewers extracted the following data from the selected studies: first author, publication year, country, setting (single-center/multicenter), study design (prospective/retrospective), number of SSPs, technical and method of SSPs resection, follow-up duration, technical success rate, AEs rate, and residual SSPs rate. Another reviewer checked all of this information for accuracy. Any conflicts or disagreements were resolved through discussion and consensus with a third author. Information about the methodological quality of each included study was recorded, and quality assessment was performed using the Newcastle-Ottawa Scale (NOS). Scores of 0–3, 4–6, and 7–9 corresponded to low, medium, and high quality, respectively [22].

### Definitions and outcomes

The primary outcome of the study was the technical success rate, which referred to the percentage of complete macroscopic resection achieved. The secondary outcomes included the rate of residual SSPs, bleeding (immediate and delayed), and perforation. The rate of residual SSPs was defined as the proportion of SSPs remaining at the resection site during the first follow-up colonoscopy. Adverse events, such as bleeding and perforation, were also recorded. Immediate bleeding rate referred to clinically significant bleeding during polyp resection that required endoscopic intervention. Delayed bleeding rate referred to clinically significant bleeding after polypectomy that required blood transfusion, radiographic embolization, or a repeat endoscopy with intervention within 14 days of polypectomy. Perforation was diagnosed by the presence of diffuse gas or intestinal fluid localized in the peritoneum. Subgroup analyses were also performed based on the technique used for SSP treatment (cold EMR vs. hot EMR), the size of the SSPs (10–19 mm vs. ≥ 20 mm), and the method of resection (en-bloc vs. piecemeal). Two independent reviewers assessed the risk of bias according to the PRISMA guidelines.

### Statistical analyses

For the meta-analysis, we analyzed proportions of patients with a 95% confidence interval (CI) using a random-effects model. To assess heterogeneity, we used two methods: Cochran’s Q test with *p* < 0.05 indicating statistically significant heterogeneity, and I^2^ statistics with values of > 50% suggesting significant heterogeneity. We conducted subgroup and sensitivity analyses to investigate possible sources of heterogeneity among the studies. Publication bias was assessed by constructing a funnel plot of each trial’s effect size against the standard error, and we evaluated funnel plot asymmetry using Egger tests with a significant publication bias defined as a *p* value < 0.05. All statistical analyses were performed using Stata version 15.0.

## Results

### Study selection and characteristics

**Fig. 1:**
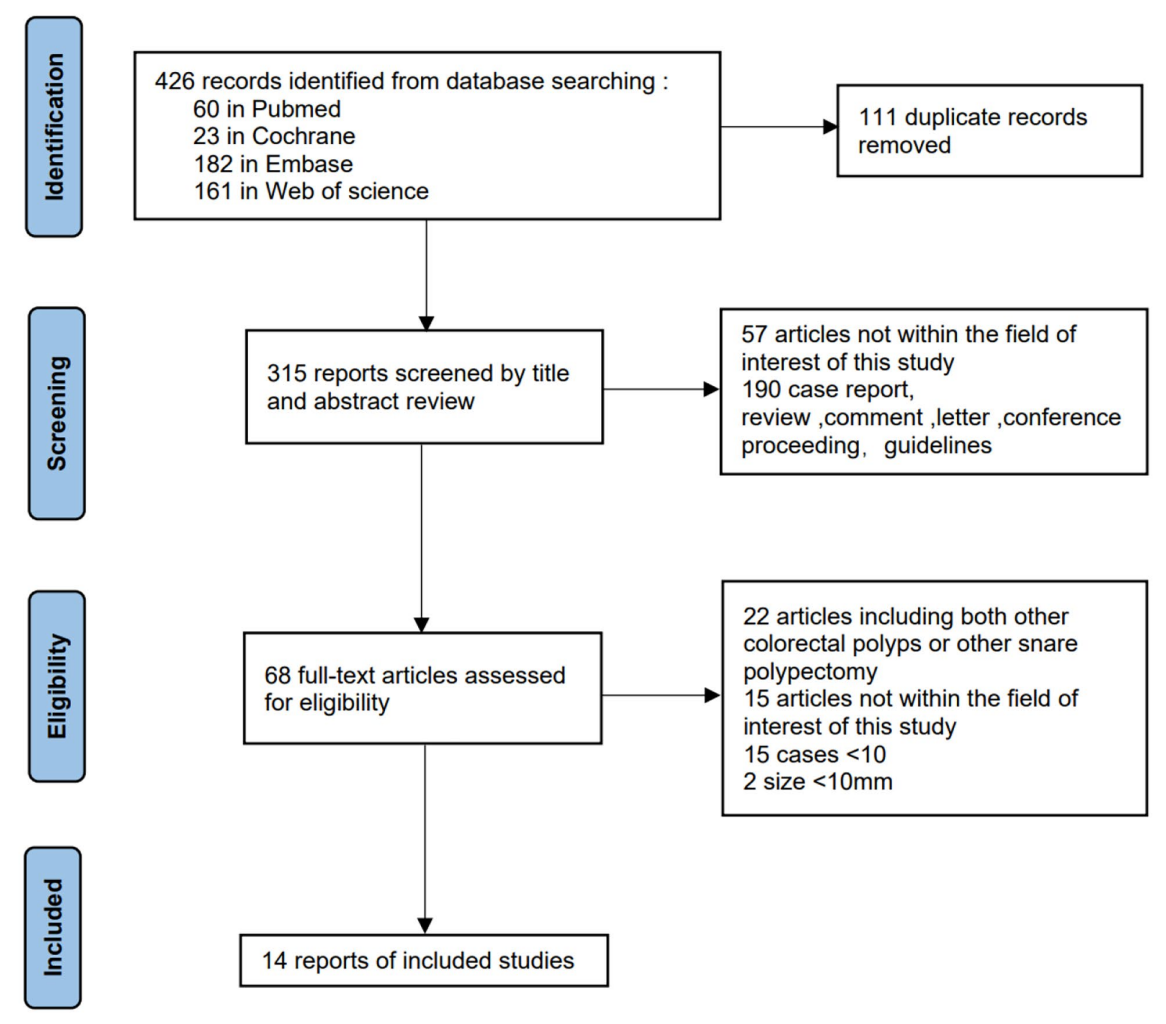
The flow diagram of the included studies

Our literature searches initially identified 426 records through database searching. After removing duplicate records (111 studies) and screening titles and abstracts (247 records), we assessed 68 full-text studies. Ultimately, 14 studies [16–21, 23–30], including 2262 SSPs, met the full inclusion criteria and were included in the final analysis (Fig. 1).

The origins of these 14 studies were as follows: seven were conducted in the USA [16, 17, 19, 23–26], five were from Australia [21, 27–30], one was from Korea [20], and one was from the UK [18]. These studies were published between 2015 and 2020. Five were prospective studies [17, 19, 27, 29, 30] and the others were retrospective studies [16, 18, 20, 21, 23–26, 28]. In addition, five studies were multi-center experiences [25, 26, 28–30] and the others were single-center experiences [16–21, 23, 24, 27]. Only SSPs removed by hot EMR or cold EMR were included in the final analysis, resulting in a total of 2262 SSPs. All studies reported techniques, with 1063 SSPs resected using cold EMR [16, 18, 19, 23, 27, 28, 30], and 1199 SSPs resected using hot EMR [17, 19–21, 24–26, 29, 30]. Eleven studies reported the method, with en-bloc resection achieved in 493 SSPs [17, 19, 24–26, 29, 30], and excision performed in 1576 SSPs [17–20, 23, 25, 26, 28–30]. Nine studies reported data on the size stratification of SSPs, with 206 SSPs being 10–19 mm in size [17, 18, 24, 27], and 1188 SSPs being ≥ 20 mm in size [17, 18, 21, 24, 26–30] (Table S2).

### Quality of included studies

The Newcastle-Ottawa Scale (NOS) was utilized to assess the methodological quality of non-randomized studies. The results indicated that 3 studies were of high quality, 8 studies were of moderate quality, and 3 studies were of low quality (Table S3).

### Primary outcome

#### Technical success rate

**Fig. 2:**
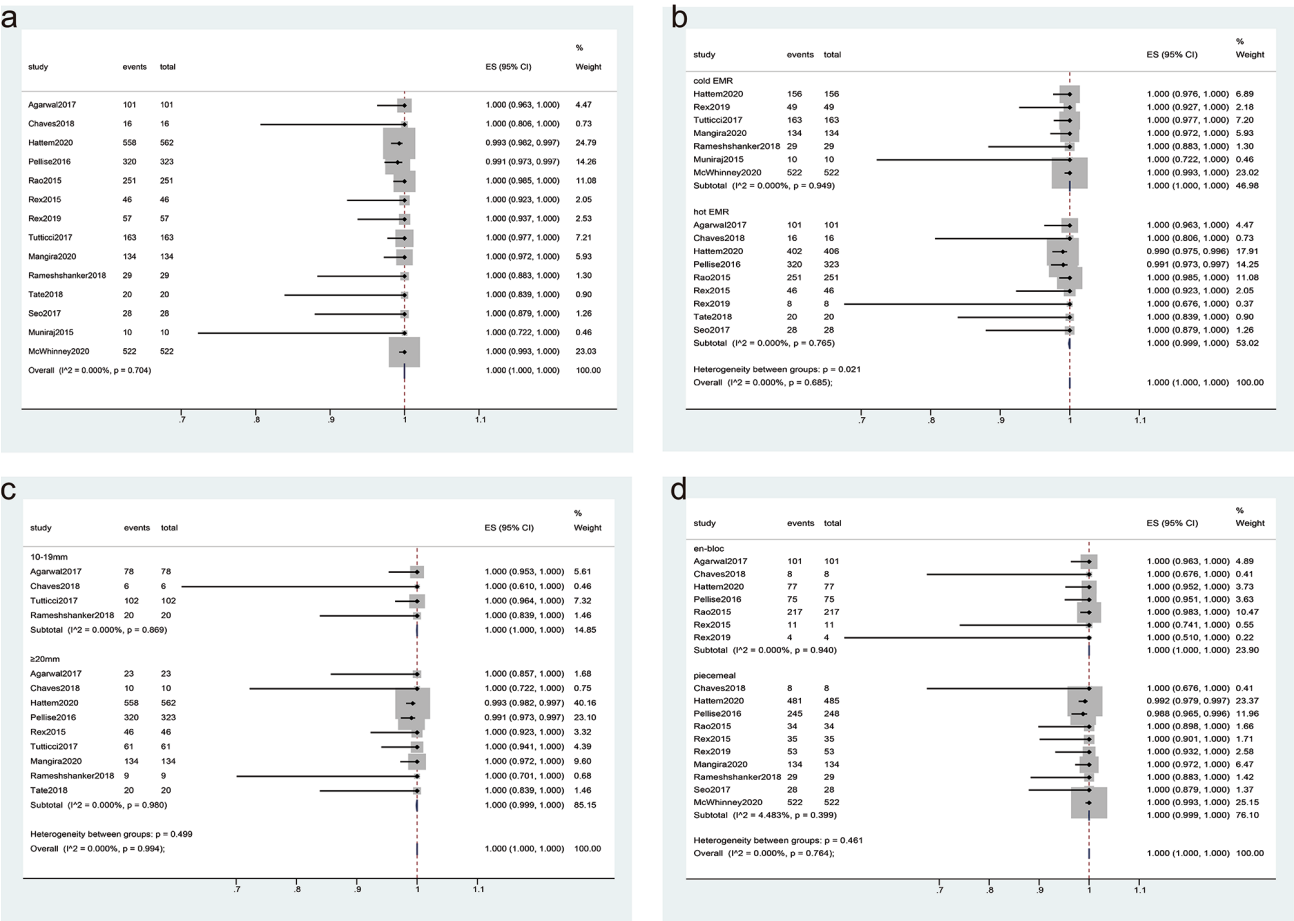
Forest plot analyzing the technique success rate for endoscopic mucosal resection (EMR). **(a)** the overall technical success rate; **(b-d)** Subgroup analyses were performed based on the technique used for sessile serrated polyps (SSPs) treatment (cold EMR vs. hot EMR) **(b)**, the size of the SSPs (10–19 mm vs. ≥20 mm) **(c)**, and the method of resection (en-bloc vs. piecemeal) **(d)**

The technical success rate was reported in all studies (Table 1). Of these 2262 SSPs, 2255 SSPs were successfully removed using EMR, resulting in an overall technical success rate of 100% (95% CI, 100–100%) with a low level of heterogeneity (I^2^ = 0%, Fig. 2a).

**Table 1:** Study Characteristics With Efficacy and safety Data

Study	techniques	methods	SSPs	SSPs followed up	Residual polyps	bleeding	Immediate bleeding	Delayed bleeding	Perforations
Agarwal2017	H	E	101	52	2	2	NA	NA	1
Chaves2018	H	E,P	16	NA	NA	0	NA	NA	NA
Hattem2020	H,C	E,P	562	399	18	23	5	18	2
Pellise2016	H	E,P	323	190	12	NA	17	14	1
Rao2015	H	E,P	251	176	5	0	0	0	0
Rex2015	H	E,P	46	46	4	NA	NA	NA	NA
Rex2019	H,C	E,P	57	NA	NA	3	2	1	0
Tutticci2017	C	NA	163	134	1	3	1	2	0
Mangira2020	C	P	134	109	4	NA	NA	NA	NA
Rameshshanker2018	C	P	29	29	1	0	NA	0	0
Tate2018	H	NA	20	9	1	NA	NA	NA	NA
Seo2017	H	P	28	28	2	NA	NA	NA	NA
Muniraj2015	C	NA	10	10	0	0	0	0	0
McWhinney2020	C	P	522	NA	NA	NA	NA	NA	NA

*H, hot EMR; C, cold EMR; E, en-bloc; P, piecemeal; NA, not available; SSPs, sessile serrated polyps

#### Subgroup analysis

Subgroup analyses were performed based on the following factors. For techniques, 1192 of 1199 SSPs were successfully removed using hot EMR, and 1063 SSPs were successfully removed using cold EMR. The technical success rate of hot EMR and cold EMR was 100% (95% CI, 99.9–100%) and 100% (95% CI, 100–100%), respectively, without significant heterogeneity (I^2^ = 0%, *P* = 0.685, Fig. 2b). The difference between subgroups was significant (*P* = 0.021).

For the size of SSPs, the technical success rate was 100% (95% CI, 100–100%) and 100% (95% CI, 99.9–100%) for sizes of 10–19 mm and ≥ 20 mm, respectively, with a low level of heterogeneity (I^2^ = 0%, Fig. 2c). The difference between subgroups was not significant (*P* = 0.499).

For the method, the technical success rate of piecemeal resection and en-bloc resection was 100% (95% CI, 99.9–100%) and 100% (95% CI, 100–100%), respectively, with a low level of heterogeneity (I^2^ = 0%, Fig. 2d). The difference between subgroups was not significant (*P* = 0.461).

#### Sensitivity analysis

The sensitivity analysis showed minimal changes in the results when each study was removed systematically of the technical success rate. This indicates that the overall conclusion regarding the technical success rate is robust and not heavily influenced by any single study (Fig S1. a).

#### Publication bias

The funnel plot and Egger test showed no publication bias for the rates of technical success (*P* = 0.199) (Fig S1. b).

### Secondary outcomes

#### Adverse events (AEs)

**Fig. 3:**
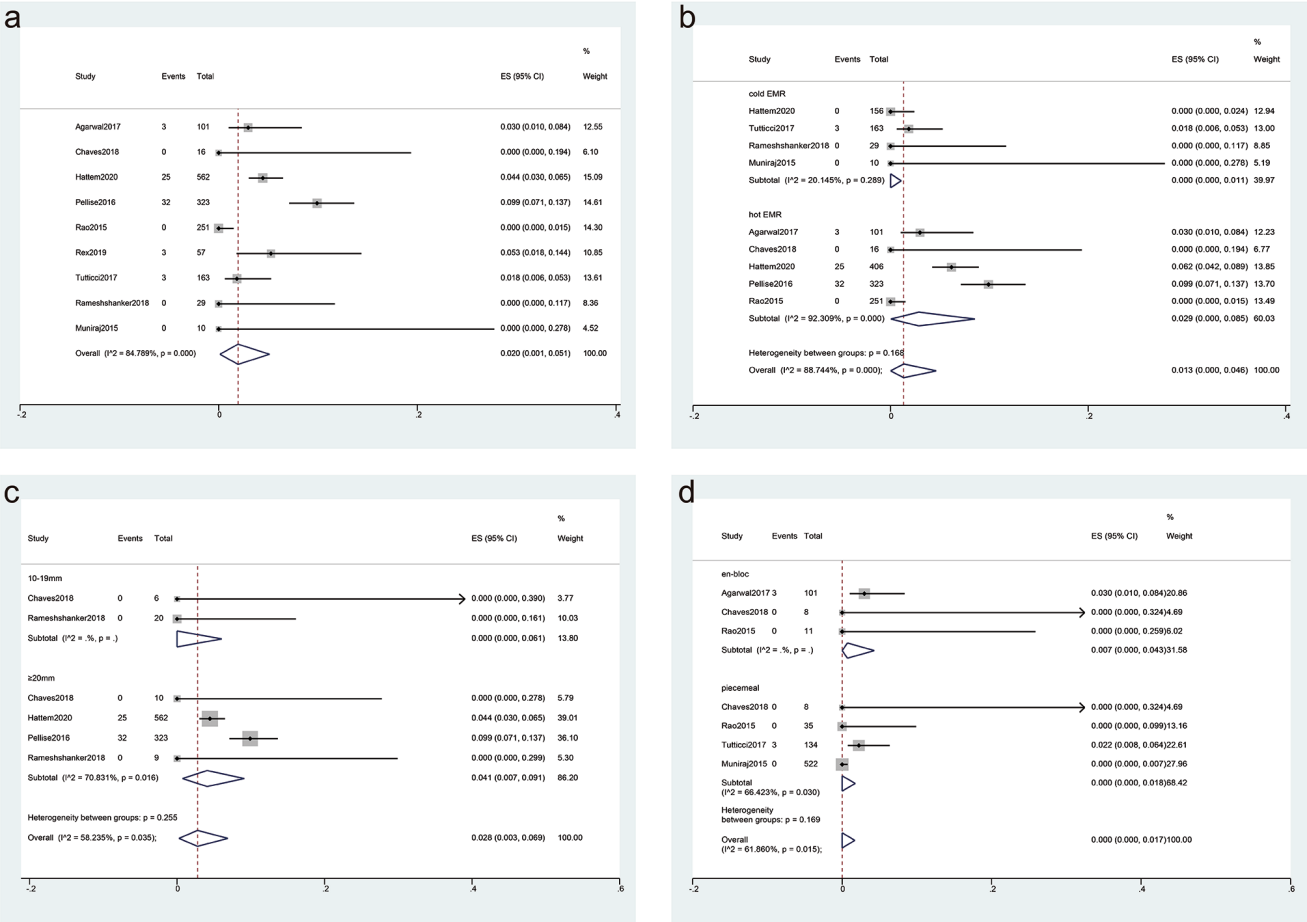
Forest plot analyzing the adverse event (AE) rate for endoscopic mucosal resection (EMR). **(a)** the overall AEs; **(b-d)** Subgroup analyses were performed based on the technique used for sessile serrated polyps (SSPs) treatment (cold EMR vs. hot EMR) **(b)**, the size of the SSPs (10–19 mm vs. ≥20 mm) **(c)**, and the method of resection (en-bloc vs. piecemeal) **(d)**

AEs were reported by nine studies for a total of 1593 SSPs (as reported in Table 1). Among these, a total of 62 cases of bleeding occurred during EMR, including 25 cases of immediate bleeding and 35 cases of delayed bleeding. In addition, 4 cases of perforation were reported. Therefore, the overall AEs rate was calculated to be 2% (95% CI, 0.1–5.1%), with a high level of heterogeneity (I^2^ = 84.79%) (Fig. 3a).

#### Subgroup analysis

A subgroup analysis (Fig. 3b) was performed based on the resection technique, which revealed that the AEs rate was 0% (95% CI, 0 − 1.1%) and 2.9% (95% CI, 0–8.5%) for cold and hot EMR, respectively. The hot EMR group exhibited a high level of heterogeneity (I^2^ = 92.31%, *P* < 0.001), but the cold EMR group did not show significant heterogeneity (I^2^ = 20.15%, *P* = 0.289), indicating that the resection technique was not the source of heterogeneity. The difference in the AE rates between the cold and hot EMR was not significant (*P* = 0.168).

Another subgroup (Fig. 3c) analysis was carried out based on the size of SSPs, which demonstrated that the AE rate was 0% (95% CI, 0 − 6.1%) and 4.1% (95% CI, 0.7–9.1%) for SSP sizes of 10–19 mm and ≥ 20 mm, respectively. As only the subgroup with SSPs ≥ 20 mm exhibited a high level of heterogeneity (I^2^ = 70.83%), suggesting the size was not considered the source of heterogeneity. The difference in the AE rates between the two size of SSPs was not significant (*P* = 0.225).

Additionally, a subgroup analysis was performed based on the resection method, revealing that the AE rate of piecemeal resection and en-bloc resection was 0% (95% CI, 0–1.8%) and 0.7% (95% CI, 0–4.3%), respectively (Fig. 3d). The difference in the AE rates between the resection method was not significant (*P* = 0.169).

In addition, the pooled rates of immediate bleeding, delayed bleeding, and perforation were 0.5%, 0.8% and 0%. Subgroup analysis revealed that the immediate bleeding rate and delayed bleeding rate were higher in the hot EMR subgroup than in the cold EMR subgroup (0.9% vs. 0%, *P* = 0.334; 1.5% vs. 0%, *P* = 0.260), and higher in SSPs > 20 mm subgroup than SSPs between 10 and 19 mm subgroup (0.7% vs. 0%, *P* = 0.763; 1.6% vs. 0%, *P* = 0.522), but the difference was not significant (Table 2).

**Table 2:** Adverse events based on size of SSPs, resection technical, and resection method

outcome	Outcomes based on size of SSP			Outcomes based on resection technical			Outcomes based on resection method			
	SSP 10-19 mm	SSP > 20 mm	*P*	Cold EMR	Hot EMR	*P*	En-bloc	piecemeal	*P*	Pooled rate
Immediate bleeding rate	0% (95% CI, 0 − 6.1%)	0.7% (95% CI, 0–5.1%; *P* = 0.002)	0.763	0% (95% CI, 0 − 0.3%; *P* = 0.730)	0.9% (95% CI, 0–4.3%; *P* = 0.000)	0.334	0% (95% CI, 0 − 9.9%)	0% (95% CI, 0–0%; *P* = 0.293)	0.369	0.5% (95% CI, 0–2.4%; *P* = 0.00)
Delayed bleeding rate	0% (95% CI, 0 − 6.1%)	1.6% (95% CI, 0.6–2.9%; *P* = 0.902)	0.522	0% (95% CI, 0 − 0.5%; *P* = 0.489)	1.5% (95% CI, 0–5.5%; *P* = 0.000)	0.260	0% (95% CI, 0 − 9.9%)	0% (95% CI, 0–0.8%; *P* = 0.100)	0.499	0.8% (95% CI, 0–2.6%; *P* = 0.00)
Perforation rate	0% (95% CI, 0 − 6.1)	0% (95% CI, 0–0%; *P* = 0.888)	0.528	0% (95% CI, 0 − 0; *P* = 0.854)	0% (95% CI, 0–0.3%; *P* = 0.594)	0.477	0% (95% CI, 0 − 1.8)	0% (95% CI, 0–0%; *P* = 0.728)	0.053	0% (95% CI,0–0; *P* = 0.91)

#### Sensitivity analysis

The sensitivity analysis demonstrated that the AEs rate was not significantly affected by systematically removing each study (Fig S2. a).

#### Publication bias

The funnel plot and Egger test indicated no evidence of publication bias for the rates of adverse events (*P* = 0.412) (Fig S2. b).

#### Residual SSPs rate

**Fig. 4:**
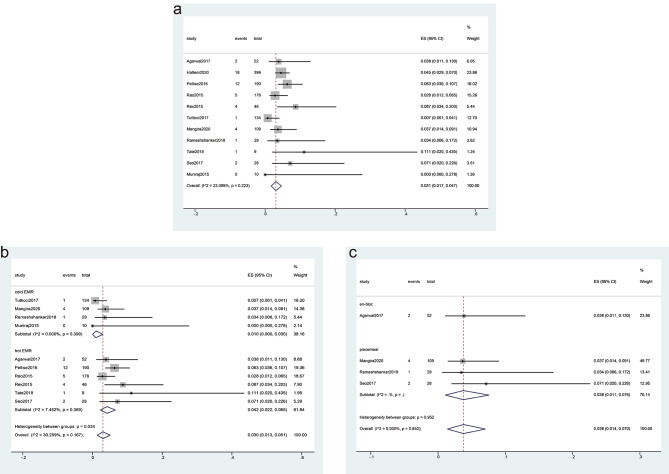
Forest plot analyzing the residual sessile serrated polyps (SSPs) rate for endoscopic mucosal resection (EMR). **(a)** the overall rate of residual SSPs; (b-c) Subgroup analyses were performed based on the technique used for SSPs treatment (cold EMR vs. hot EMR) **(b)**, and the method of resection (en-bloc vs. piecemeal) **(c)**

11 studies reported the rate of residual SSPs, and there were 50 residual SSPs for 1182 SSPs with a follow-up duration ranging from 2 to 74 months. The overall rate of residual SSPs was 3.1% (95% CI, 1.7–4.7%) with a low level of heterogeneity (I^2^ = 23.1%) (Fig. 4a).

### Subgroup analysis

We conducted subgroup analyses based on the techniques used for EMR and the methods of SSPs resection. The results showed that the residual rate was significantly lower with cold EMR (1.0%; 95% CI, 0–3.0%) than with hot EMR (4.2%; 95% CI, 2.2–6.5%), with a low level of heterogeneity (I^2^ = 30.3%, Fig. 4b). The difference between the rates was statistically significant (*P* = 0.034). Regarding the method of SSPs resection, the subgroup analysis revealed a residual rate of 3.8% (95% CI, 1.1–13%) for en-bloc EMR and 3.8% (95% CI, 1.1–7.6%) for piecemeal EMR, with a low level of heterogeneity (I^2^ = 0.0%, Fig. 4c). The difference between the rates was not significant (*P* = 0.952).

#### Sensitivity analysis

We performed a sensitivity analysis to evaluate the robustness of our findings. We systematically removed each study and assessed the resulting changes in sensitivity. The analysis showed little sensitivity change, indicating the stability of our findings (Fig S3. a).

#### Publication bias

We conducted a funnel plot analysis and Egger’s test to assess publication bias. The results indicated no significant publication bias (*P* = 0.669) (Fig S3. b).

## Discussion

In a recent meta-analysis of cold EMR resections of ≥ 10 mm SSPs, the literature was searched until January 2021 only [8]. Therefore, inclusion of updated clinical randomized controlled studies and larger sample sizes for systematic analysis of the efficacy and safety of cold EMR for resection of ≥ 10 mm SSPs is warranted. Thus, we conducted a systematic review to provide a comprehensive analysis of the outcomes associated with the removal of SSPs using EMR. We also aimed to compare the outcomes of cold EMR vs. hot EMR, SSPs size of 10–19 mm vs. ≥20 mm, and SSPs resection method of en-bloc vs. piecemeal. Our meta-analysis showed a high technical success rate of 100% and a low adverse event rate of 2%, as well as a low residual SSPs rate of 3.1%. Subgroup analysis revealed that the rates of adverse events were lower in cold EMR compared to hot EMR (0% vs. 2.9%, *P* = 0.168), in polyps that were 10–19 mm compared to those ≥ 20 mm (0% vs. 4.1%, *P* = 0.255), and in en-bloc resection compared to piecemeal resection (0% vs. 0.7%, *P* = 0.169). The residual SSPs rate was significantly lower in cold EMR than in hot EMR (1.0% vs. 4.2%, *P* = 0.034).

In our study, a total of 2262 SSPs were included, with 1063 SSPs resected using cold EMR and 1199 SSPs resected using hot EMR. In contrast, Chandrasekar reported data on 1137 SSPs, with 793 resected using hot EMR, 195 resected using cold EMR, and 149 removed via snare polypectomy (108 with a hot snare and 41 with a cold snare) [11]. Li et al. [12] conducted a meta-analysis to assess the effectiveness and safety of cold snare endoscopic resection for SSPs that were 10 mm or larger. The overall technical success rate was 99.5%, with AEs occurring in 2.7% of cases and residual SSPs observed in 4.3% of cases. Out of the 1727 SSPs, 713 were resected using cold snare, and 1014 were resected using cold EMR. The technical success rate was 100%, with AEs occurring in only 0.7% of cases and residual SSPs observed in 2.9% of cases. These findings demonstrate that our analysis not only had the largest sample size but also included the most SSPs resected using cold EMR or hot EMR compared to other studies. Thus, it can be concluded that SSPs can be safely and efficiently removed using EMR.

Previous studies have reported similar technical success rates for cold and hot EMR when removing SSPs. The immediate bleeding rate for cold EMR has been reported as 4.6%, while for hot EMR it ranges from 2.2 to 6.7% for all polyps. Additionally, cold EMR-related adverse events have been shown to be significantly lower (between 0% and 0.5%) compared to hot EMR (5%) [11, 12, 31, 32]. These findings suggest that cold EMR may be a safer alternative to hot EMR for the removal of SSPs [33]. It is crucial to acknowledge that current guidelines strongly advocate for the complete removal of small polyps through CSP. However, the guidelines do not recommend the use of CSP for large polyps due to the likelihood of residual tissue remaining after surgery. As a result, hot resection techniques have traditionally been the preferred method for removing large polyps [11]. But between the adverse effects associated with electrocoagulation, researchers are constantly improving both resections [33]. In the subgroup analysis of this study, we found that the rates of technical success were comparable between cold EMR and hot EMR, both achieving 100% success rates. However, cold EMR had a significantly lower residual rate than hot EMR, with a rate of 1.0% compared to 4.2% (*p* = 0.034), and a slightly lower AE rate, with no reported cases compared to 2.9% for hot EMR. In our pooled analysis, we also found that both immediate and delayed bleeding rates were lower in cold EMR than in hot EMR, with rates of 0% compared to 0.9% and 0% compared to 1.5%, respectively. These differences may be attributed to the use of electrocautery in hot EMR, which can create a deeper resection plane and increase the risk of transecting thicker blood vessels in the submucosal layers, leading to unintended transmural capture. Overall, our results suggest that cold EMR is superior to hot EMR, with similar technical success rates and lower rates of AEs and residual SSPs.

Bronsgeest et al. [32] reported the residual polyp rate for polyps > 20 mm to be 18.8% after conventional EMR. The previous studies that focused solely on SSPs showed that the residual rate for residual SSPs of ≥ 10 mm and ≥ 20 mm was 2.9% and 4.7–5.9%, respectively. These rates are comparable to our pooled residual rate of 3.1% and 4.1% for all SSPs and those ≥ 20 mm, respectively [11, 12]. Previous pooled analyses have reported an incidence of overall delayed bleeding rates ranging from 1.1 to 1.3% for SSPs > 10 mm and 1.7–2% for SSPs > 20 mm. However, our pooled cohort demonstrated a lower residual rate for all SSPs, with a rate of 3.1% for SSPs of all sizes and 4.1% for SSPs ≥ 20 mm. The AE and residual rates were slightly higher in SSPs > 20 mm compared to SSPs between 10 and 19 mm, but still within the acceptable level [25].

En-bloc EMR offers a significant advantage over piecemeal EMR in that it provides a more reliable histologic interpretation of the resected specimen at the tissue margins. Our study found that en-bloc resection was associated with a lower residual polyp rate compared with piecemeal resection (2.3% vs. 4%) [34]. Our study found that en-bloc resection had a comparable residual rate to piecemeal resection for subcentimeter polyps (SSPs). It has been reported that SSPs tend to lift easily with submucosal injection due to their relatively loose attachment to the deep submucosa and are also not associated with significant submucosal fibrosis, which allows for better resections compared to conventional adenomas [29].

It is important to acknowledge the limitations of our analysis. The majority of studies included in the meta-analysis were retrospective and conducted at a single center, with varying durations of follow-up. Moreover, there is a lack of published randomized controlled trials on this topic. Additionally, the data were not available to assess the residual rate based on the size of the SSPs, which may limit the generalizability of our findings. The loss of a few patients during follow-up and the variable follow-up duration (ranging from 2 to 74 months) may also have impacted the results. Finally, it should be noted that there was no standardized technique used across the studies to identify residual tissue at the polypectomy site. Hence, it is imperative to establish precise clinical protocols, including specifications such as the type of endoscope, snare, electrical current, submucosal injection solution, utilization of auxiliary tools, and the expertise of endoscopists, in forthcoming research endeavors. Additionally, comprehensive long-term data on polyp recurrence should be established to facilitate thorough comparisons of the merits and drawbacks of various techniques. Furthermore, an increased number of randomized controlled trials are necessary to yield more definitive evidence regarding the optimal approach to sub-centimeter polypectomy.

## Conclusion

According to the literature review conducted in this investigation, it is evident that cold EMR demonstrates superior therapeutic efficacy and reduced incidence of adverse reactions when the submucosal space is cleared by ≥ 10 mm. This suggests that cold EMR is a reliable and efficient procedure, although further substantiation through extensive clinical trials is necessary.

### Electronic supplementary material

Below is the link to the electronic supplementary material.

Supplementary Material 1

## Data Availability

All data related to this study are included in this published article and its supplementary information files. please send mail to Xiaofeng Zhang for further information.
