# Supplementary material for: Cold EMR vs. Hot EMR for the removal of sessile serrated polyps larger than 10 mm: a systematic review and meta-analysis
Source: BMC Surg. 2024 Mar 20;24:93. doi: 10.1186/s12893-024-02325-2 (PMC10953062; doi:10.1186/s12893-024-02325-2)
Supplement: Supplementary file 1 — Supplementary Material 1 [file 12893_2024_2325_MOESM1_ESM.docx]

**Supplementary Tables**

| Table S1. Search Strategy on PubMed Electronic Database | |  |
| --- | --- | --- |
| Search | Query | Items found |
| #3 | #1 AND #2 | 60 |
| #2 | (((((((sessile serrated adenoma[MeSH Terms]) OR (sessile serrated polyp[MeSH Terms])) OR (sessile serrated lesion[MeSH Terms])) OR (SSA[MeSH Terms])) OR (SSP[MeSH Terms])) OR (SSA/P[MeSH Terms])) OR (SSL[MeSH Terms])) OR (((((((sessile serrated adenoma[Title/Abstract]) OR (sessile serrated polyp[Title/Abstract])) OR (sessile serrated lesion[Title/Abstract])) OR (SSA[Title/Abstract])) OR (SSP[Title/Abstract])) OR (SSA/P[Title/Abstract])) OR (SSL[Title/Abstract])) | 26702 |
| #1 | ("Endoscopic Mucosal Resection"[Mesh]) OR (((((((((((((Endoscopic Mucosal Resections[Title/Abstract]) OR (Mucosal Resection, Endoscopic[Title/Abstract])) OR (Resection, Endoscopic Mucosal[Title/Abstract])) OR (Strip Biopsy[Title/Abstract])) OR (Biopsy, Strip[Title/Abstract])) OR (Strip Biopsies[Title/Abstract])) OR (Endoscopic Mucous Membrane Resection[Title/Abstract])) OR (Endoscopic Submucosal Dissection[Title/Abstract])) OR (Dissection, Endoscopic Submucosal[Title/Abstract])) OR (Endoscopic Submucosal Dissections[Title/Abstract])) OR (Submucosal Dissection, Endoscopic[Title/Abstract])) OR (Endoscopic Full Thickness Resection[Title/Abstract])) OR (Submucosal Tunneling Endoscopic Resection[Title/Abstract])) | 8573 |

| Table S2. Baseline Characteristics | | | | | | | | | | |
| --- | --- | --- | --- | --- | --- | --- | --- | --- | --- | --- |
| Study | Country | Centers | Study  design | Total SSPs (n) | techniques | | Method | | Size | |
|  |  |  |  |  | Hot EMR | Cold EMR | en-bloc | piecemeal | 10-19 mm | ≥20mm |
| Agarwal2017 | USA | 1 | retrospective | 101 | 101 | 0 | 101 | 0 | 78 | 23 |
| Chaves2018 | USA | 1 | prospective | 16 | 16 | 0 | 8 | 8 | 6 | 10 |
| Hattem2020 | Australia | 4 | prospective | 562 | 406 | 156 | 77 | 485 | 0 | 562 |
| Pellise2016 | Australia | 8 | prospective | 323 | 323 | 0 | 75 | 248 | 0 | 323 |
| Rao2015 | USA | 2 | retrospective | 251 | 251 | 0 | 217 | 34 | NA | NA |
| Rex2015 | USA | 2 | retrospective | 46 | 46 | 0 | 11 | 35 | 0 | 46 |
| Rex2019 | USA | 1 | prospective | 57 | 8 | 49 | 4 | 53 | NA | NA |
| Tutticci2017 | Australia | 1 | prospective | 163 | 0 | 163 | NA | NA | 102 | 61 |
| Mangira2020 | Australia | 5 | retrospective | 134 | 0 | 134 | 0 | 134 | 0 | 134 |
| Rameshshanker2018 | United Kingdom | 1 | retrospective | 29 | 0 | 29 | 0 | 29 | 20 | 9 |
| Tate2018 | Australia | 1 | retrospective | 20 | 20 | 0 | NA | NA | 0 | 20 |
| Seo2017 | Korea | 1 | retrospective | 28 | 28 | 0 | 0 | 28 | NA | NA |
| Muniraj2015 | USA | 1 | retrospective | 10 | 0 | 10 | NA | NA | NA | NA |
| McWhinney2020 | USA | 1 | retrospective | 522 | 0 | 522 | 0 | 522 | NA | NA |
| Total |  |  |  | 2262 | 1199 | 1063 | 493 | 1576 | 206 | 1188 |
| NA, not available; | | | | | | | | | | |

| Table S3. Quality assessment of studies included using the Newcastle-Ottawa Scale | | | | |
| --- | --- | --- | --- | --- |
| Study | Selection | Comparability | Outcome | Total score |
| Agarwal2017 | 3 | 2 | 2 | 7 |
| Chaves2018 | 2 | 0 | 1 | 3 |
| Hattem2020 | 2 | 0 | 3 | 5 |
| Pellise2016 | 4 | 0 | 2 | 6 |
| Rao2015 | 3 | 0 | 3 | 6 |
| Rex2015 | 3 | 0 | 3 | 6 |
| Rex2019 | 4 | 0 | 3 | 7 |
| Tutticci2017 | 2 | 0 | 2 | 4 |
| Mangira2020 | 2 | 0 | 1 | 3 |
| Rameshshanker2018 | 2 | 0 | 2 | 4 |
| Tate2018 | 2 | 0 | 2 | 4 |
| Seo2017 | 4 | 2 | 2 | 8 |
| Muniraj2015 | 2 | 0 | 1 | 3 |
| McWhinney2020 | 2 | 0 | 2 | 4 |

**Supplementary figure legends**


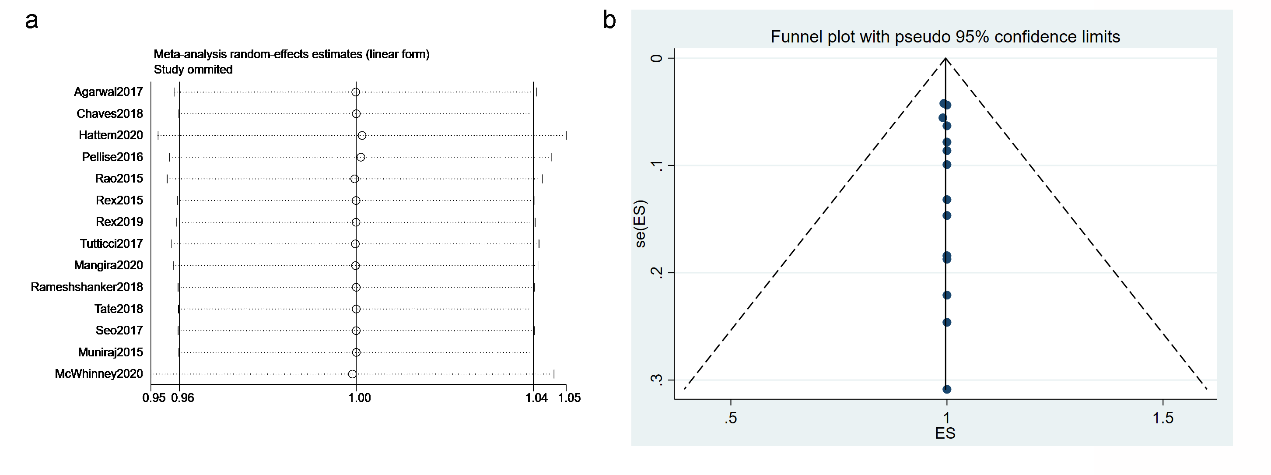


Fig S1. a, Sensitivity Analysis for Technical success rate; b, Publication bias for technical success (*p* = 0.199)


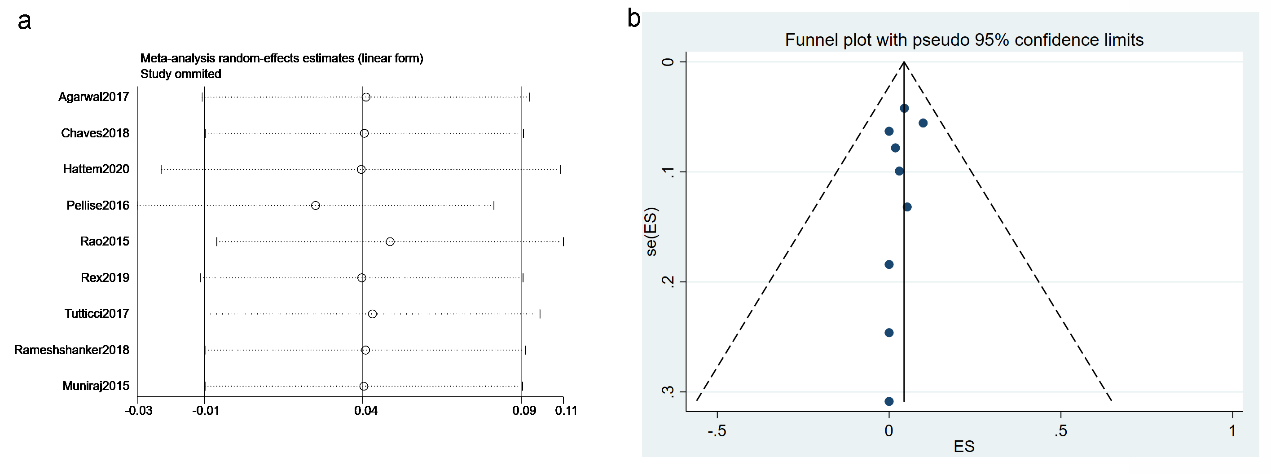


Fig S2. a, Sensitivity Analysis for adverse event rate; b, Publication bias for adverse event rate (*p* = 0.412)


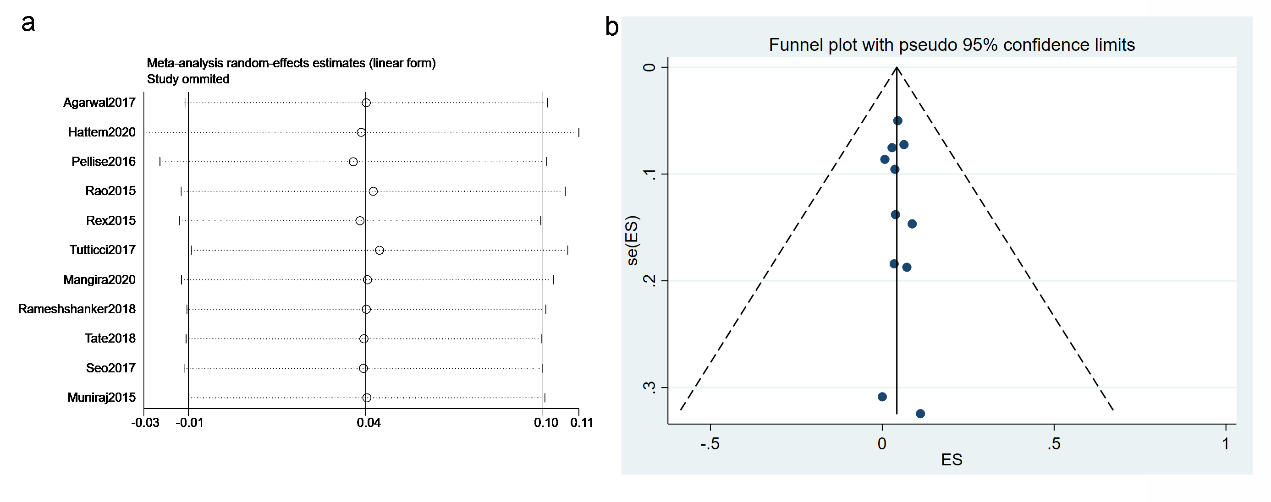


Fig S3. a, Sensitivity Analysis for residual SSPs rate; b, Publication bias for residual SSPs rate (*p* = 0.699)
